# A calculated risk: Evaluating HIV resistance to the broadly neutralising antibodies10-1074 and 3BNC117

**DOI:** 10.1097/COH.0000000000000764

**Published:** 2022-09-27

**Authors:** Panagiota Zacharopoulou, M. Azim Ansari, John Frater

**Affiliations:** a Peter Medawar Building for Pathogen Research, Nuffield Department of Medicine, University of Oxford; b NIHR Oxford Biomedical Research Centre, Oxford, UK

**Keywords:** 10-1074, 3BNC117, broadly neutralising antibody, HIV, resistance

## Abstract

Broadly neutralising antibodies (bNAbs) are a promising new therapy for the treatment of HIV infection. However, the effective use of bNAbs is impacted by the presence of preexisting virological resistance and the potential to develop new resistance during treatment. With several bNAb clinical trials underway, sensitive and scalable assays are needed to screen for resistance. This review summarises the data on resistance from published clinical trials using the bNAbs 10-1074 and 3BNC117 and evaluates current approaches for detecting bNAb sensitivity as well as their limitations.

**Recent findings:**

Analyses of samples from clinical trials of 10-1074 and 3BNC117 reveal viral mutations that emerge on therapy which may result in bNAb resistance. These mutations are also found in some potential study participants prior to bNAb exposure. These clinical data are further informed by ex-vivo neutralisation assays which offer an alternative measure of resistance and allow more detailed interrogation of specific viral mutations. However, the limited amount of publicly available data and the need for better understanding of other viral features that may affect bNAb binding mean there is no widely accepted approach to measuring bNAb resistance.

**Summary:**

Resistance to the bNAbs 10-1074 and 3BNC117 may significantly impact clinical outcome following their therapeutic administration. Predicting bNAb resistance may help to lower the risk of treatment failure and therefore a robust methodology to screen for bNAb sensitivity is needed.

## INTRODUCTION

The discovery and isolation of broadly neutralising antibodies (bNAbs) in the late 2000s [1,2] was a breakthrough with great promise for HIV prevention [3,4] and cure [5]. These antibodies are found only in a rare subpopulation of people living with HIV (PWH) [6]. Unlike strain-specific neutralising antibodies (NAbs), which arise in the majority of PWH, bNAbs can neutralise a wide range of viral strains circulating in the host by targeting multiple variants of the HIV Envelope protein (Env) [7]. bNAb neutralisation occurs through inhibiting different mechanisms of viral entry, such as steric interference with viral attachment to the cell, immobilisation of the Env trimer or acceleration of Env spike decay [8]. In clinical trials [9–15] passive administration of bNAbs significantly reduces viremia in participants who are not taking antiretroviral therapy (ART) and can maintain viral suppression in those on ART who subsequently undergo a treatment interruption. However, longer term viral suppression may be compromised due to the selection of preexisting resistant viruses emerging from the latent reservoir or the selection of newly formed escape variants generated by sub-optimal selection pressure [16]. Using dual rather than monotherapy with bNAbs helps overcome some issues around resistance and substantially extends the duration of viral remission in those stopping ART [13]. However, even when using two bNAbs together, resistance remains a barrier that needs to be removed to achieve truly long-term drug-free remission. In this article, we will review two of the most promising bNAbs, 3BNC117 and 10-1074, aiming to understand how bNAb resistance emerges, what its effect on viral fitness and functionality might be, and how best to measure it. 

**Box 1:**
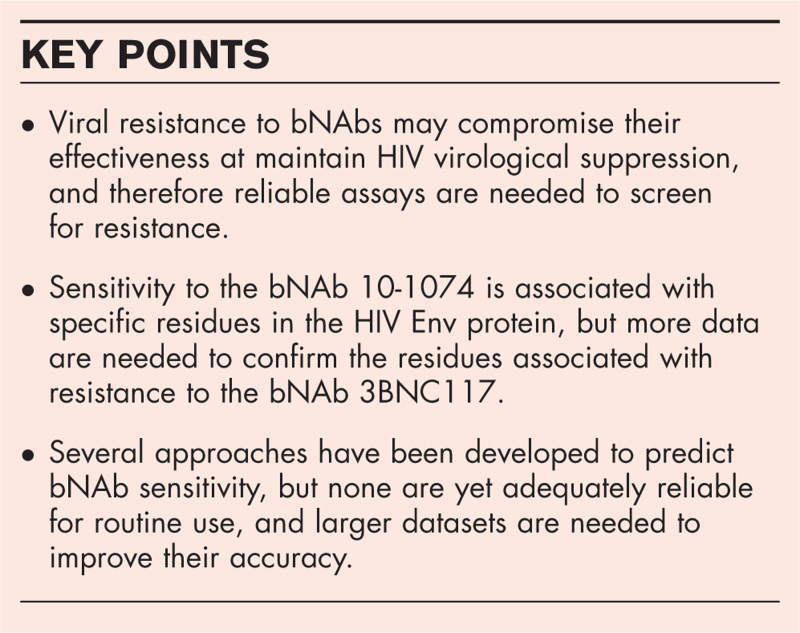
no caption available

## 10-1074 AND 3BNC117 IN THE CLINIC: ESCAPE SIGNALS

Passive administration of 10-1074 and 3BNC117 – generally intravenously - alone or in combination is being tested as an HIV treatment in an increasing number of clinical trials [11–13,15,17,18^▪▪^], with hope for long-term drug-free remission, and even cure. Single infusions of 10-1074 or 3BNC117 in viraemic participants have been shown to transiently reduce viremia in two clinical trials; [10,12] however, sustained or full viral suppression was not achieved. BNAb resistant clones were selected for in all participants that received 10-1074 monotherapy and in the majority of those who received 3BNC117. In an antiretroviral treatment interruption (ATI) setting - with participants who were screened for bNAb sensitivity -infusions of 3BNC117 resulted in longer periods of undetectable viraemia compared to historical controls (average of 6.7 and 9.9 weeks of viral suppression off ART depending on the number of doses, vs. 2.9 weeks in controls) [15]. Moreover, a few participants did not rebound as long as bNAb levels remained therapeutic, which suggests that adequate serum levels of 3BNC117 may prevent the development or selection of escape mutations [15]. However, a 2018 study [14] showed that when 3BNC117 was administered in PWH who underwent ATI, preexisting resistance was a strong predictor of shorter time to viral rebound [14].

As has been learned from the field of antiretroviral therapy, one approach to overcoming resistance is to give drugs together in combination. The joint administration of 10-1074 and 3BNC117followed by ART interruption in PWH with bNAb sensitive viruses mediated viral suppression for an extended period (median 21 weeks) [13] – a longer period than monotherapy – and supporting the argument for combination therapy. These findings were confirmed in the most recent phase 1b clinical trial of 10-1074 and 3BNC117 combination given to PWH on or off antiretroviral treatment [18^▪▪^]. Of note, when administered to viraemic participants, the combination of 3BNC117 and 10-1074 was not as effective; although a reduction in viremia was observed, full viral suppression was only seen in one participant with a low baseline viral load [11]. This suggests that for bNAbs to be fully effective, viral suppression may first need to be achieved with ART. Only participants with chronic infection were recruited in the clinical trials mentioned above, and it is that treatment in primary infection is more effective due to smaller HIV reservoirs and lower viral diversity.

**FIGURE 1:**
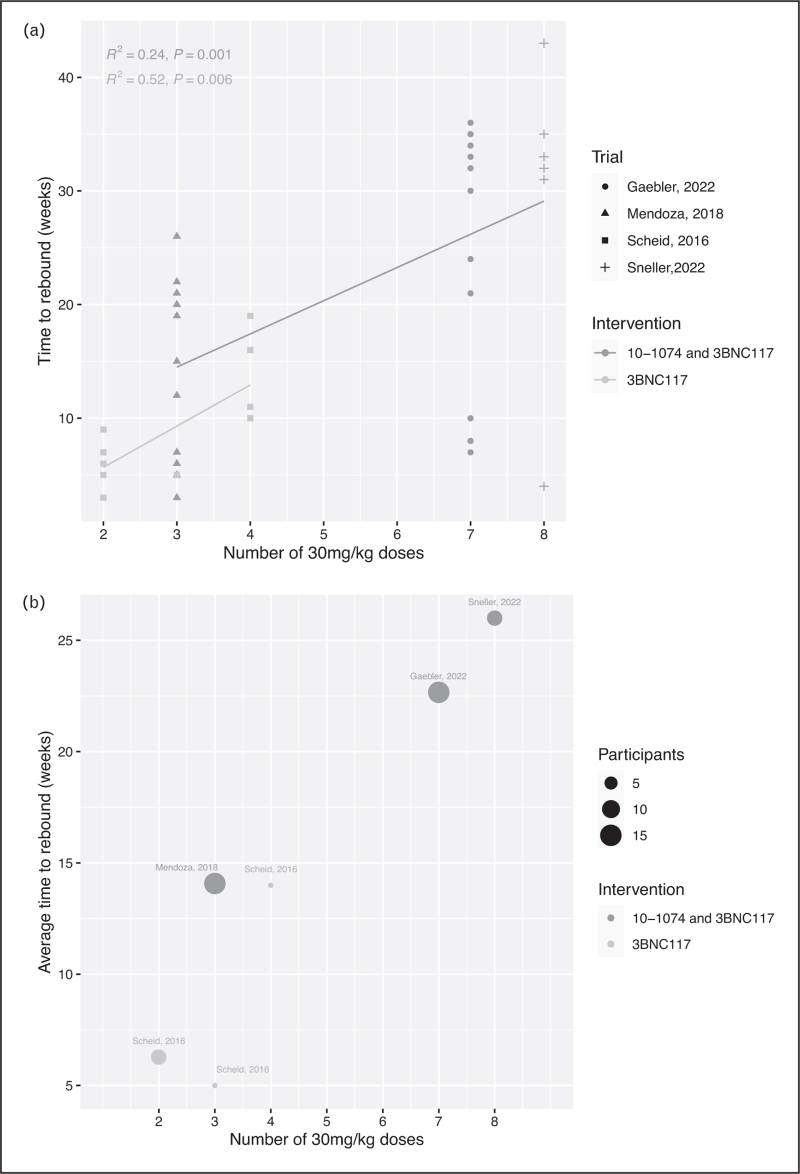
Average time to viral rebound after treatment with 10-1074 and 3BNC117 combination or 3BNC117 alone, in participants post ART interruption. a. Each data point represents a study [13,15,18^▪▪^,^19^^▪▪^] and their size reflects the number of participants in each arm. b. Individual study participants’ time to rebound data points are shown and each study cohort [13,15,18^▪▪^,^19^^▪▪^] is represented by a different shape. A fitted line per group (combination of 10-1074 and 3BNC117, and 3BNC117 monotherapy shows the association of number of doses with time to viral rebound. The participants in these studies received different number of bNAb doses (30 mg/kg). Time to viral rebound in weeks is shown on y-axis and number of bNAb doses is shown on x-axis.

The longer the duration for which bNAbs are above therapeutic levels, the greater the resulting period of viraemic control. Figure 1 shows summary data from currently published clinical trials and shows that increasing numbers of doses of 3BNC117 alone or combined with 10-1074, result in longer periods of control in participants who interrupted ART immediately after receiving bNAbs (Fig. 1A, B). Cox regression analysis using combined data from studies that report both baseline sensitivity and time to viral rebound [13,18^▪▪^,^19^^▪▪^] showed a 37% decrease in viral rebound per increase in number of bNAb doses (hazard ratio, HR: 0.63, 95% confidence interval, CI: 0.50-0.78, *p*-value = 0.00004). Also, presence of resistance at baseline was associated with a 44% increase in expected viral rebound relative to absence of resistance (HR: 1.44, 95% CI: 0.60-3.45, *p*-value = 0.4), although this effect was not statistically significant.(The times to viral rebound for Sneller *et al.*[19^▪▪^] are sampled by approximation from the paper figures.) One would expect, however, that these data will be strengthened by the results of clinical trials using long-acting ‘LS’ bNAb variants, which may result in 4-6 months suppression after single dosing.

## BROADLY NEUTRALISING ANTIBODIES TARGETING EPITOPES AND ESCAPE MUTATIONS

Common features of bNAbs include extensive somatic hypermutation, the ability to recognise N-glycans as epitopes and long heavy chain complementary determining regions (CDRs) that reach protein epitopes concealed by the glycan shield or other Env protein loops. BNAbs target conserved HIV Env sites, which include the CD4 binding site (CD4bs), the V3 glycan site, the V2 apex, the MPER site and the gp120-gp41 interface [20]. Notably, several bNAb epitopes, such as V3 and the CD4bs, have been shown to serve as strain-specific Nab targets [21].

10-1074 is an HIV Env V3 loop-targeting bNAb that interacts with the glycan attached on the potential N-glycosylation site (PNGS) at position 332 and binds the underlying ^324^GDIR^327^ motif [12,22–24], which mediates CCR5 binding. The significance of this amino acid position was demonstrated by Caskey *et al*. [12], who observed that the 2 out of 19 PWH who had single N332T and D325E mutations in 100% of their preinfusion plasma viruses, did not respond to 10-1047 monotherapy. Interestingly, different residues at a given position are thought to affect 10-1074 escape heterogeneously. For instance, a serine or asparagine replacement of D325 on the BG505 viral strain was found to have no effect on 10-1074 binding, unlike D325E [25]. However, an intact PNG does not guarantee the presence of a glycan. Studies have indicated that the probability of glycan occupation was associated with the amino acid at the second position in the PNG sequon [26]. In their paper looking at how V3 bNAbs bind on the GDIR motif, Sok *et al.* showed that the absence of the glycan on either position 332 or 334 is associated with higher frequency of mutation atD325, R327 or H330 residues, which might affect bNAb neutralisation [23].

3BNC117 is a VRC01-class bNAb that mimics CD4 binding [27] CD4 binding site bNAb epitopes need to be conserved and lie within an HIV Env cavity, protected by the Env V1V2 and V3 regions and the glycan shield [28]. The contact residues of 3BNC117 on Env are located in Loop D, the CD4 binding loop and the β23 V5 loop (HXB2 amino acid positions 274–283, 364–374 and 455–471, respectively) [9,14]. Studies indicate that mutations emerging both inside and outside the known CD4 binding site contact residues are associated with VRC01-class bNAb resistance and may confer different replication fitness costs, compensating each other to overcome resistance [29,30^▪^]. In particular, mutations G459D, Q363H, S461D, and S274Y have been associated with different levels of 3BNC117 resistance due to bNAb binding interference [10]. These resistance mutations were, however, not observed in the rebound viruses of PWH who received 3BNC117 infusions; R456S and atypical residues in K272 were found in the majority of rebound viruses in two participants, which may affect the structure of V5 and disrupt 3BNC117 binding [15]. A mutation in residue 459, where glycine was replaced by isoleucine, was also detected in 100% of rebound viruses, although 459I was present in a minority of pre3BNC117 infusion plasma viruses in PWH [14]. Notably, no single mutations that affect the sensitivity of both bNAbs were detected in clinical trials where a combination of 3BNC117 and 10-1074 was administered in PWH [11,13]. Intrinsic bNAb sensitivity may also differ among HIV subtypes. For example, 3BNC117 is less inhibitory against C clade viruses, and 10-1074 is less effective against CRF01-AE, A and C viruses due to either complete lack or under-representation of the 332 PNGS [29].

Structural changes caused by insertions and/or deletions in the hypervariable HIV Env regions may also mediate bNAb escape. Some studies propose that the main role of the HIV V1 and V2 Env regions is as a defence mechanism against the host immune response [31]. Concordant with this, increased V1 and V2 length correlates with V3 and CD4bs bNAb resistance [29,32,33]. Changes in the length of the HIV Env V4 region have been reported to affect the structure of the glycan shield that surrounds the CD4 binding site [34]. A longer V5 loop has also been associated with 3BNC117 resistance, due to steric Env changes that cause clashing with the bNAb heavy and light chains [10,13]. Moreover, high viral diversification during untreated infection is thought to be more likely to give rise to bNAb escape mutations [35^▪^], especially within the neutralising antibody epitopes that overlap with bNAb binding sites [36].

**FIGURE 2:**
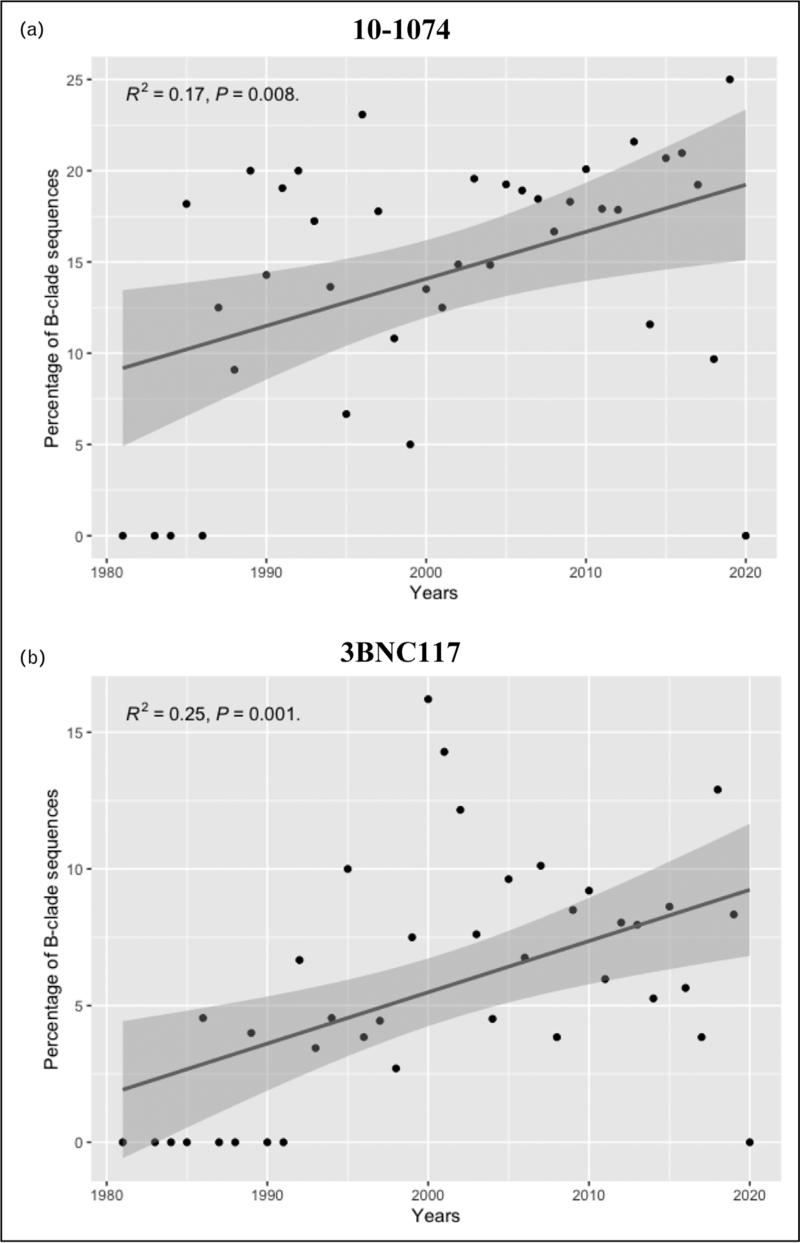
BNAb sensitivity in B clade env sequences across time, based on a bNAb sensitivity prediction algorithm. Resistance is defined based on the detection of mutations associated with a.10-1074 resistance (HXB2 sites: 325, 330, 332, 333 and 334), b. 3BNC117 resistance (HXB2: 279, 280, 456, 457, 458, 459) [13]. Each point represents the percentage of resistant sequences, and the grey ribbon is the confidence interval.

Lastly, it has been proposed that the adaptation of HIV to humoral responses over time may lead to increased resistance to neutralisation by bNAbs at a population level, and across time and different clades [37]. To explore this further we ran a bNAb sensitivity prediction algorithm [18^▪▪^]. on B clade *env* protein sequences downloaded from the los Alamos database (https://www.hiv.lanl.gov/) which had been collected from PWH since the beginning of the pandemic (2816 sequences). For each year since 1986, we estimated the number of sequences that carried residues associated with resistance to 10-1074. Interestingly, the analysis reveals a trend of increasing frequency of residues associated with 10-1074 resistance over time (*R*^2^=0.17, *P* < 0.05) (Fig. 2A). A similar trend of increasing resistance to 3BNC117 over time was observed in the dataset using the same algorithm (Fig. 2B), although it should be noted that the algorithm for 3BNC117 may be less reliable at predicting resistance.

As bNAbs have only recently become available as a therapy, this would suggest that overlapping pressures from circulating antibodies may result in immune escape, which is potentially transmitted to new hosts resulting in an increase in population level resistance. Should this pattern continue at the current rate – or even accelerate – this may have future implications for the broader utility of bNAb therapy.

## LIMITATIONS OF CURRENT BROADLY NEUTRALISING ANTIBODY SENSITIVITY SCREENING ASSAYS. WHAT DOES THE FUTURE HOLD?

Currently, the gold standard for assessing bNAb resistance in viral strains is the in-vitro neutralisation assay [15], a process that is resource- and time-expensive but also potentially difficult to interpret clinically [13,15,16]. Epitope mapping for 10-1074 and 3BNC117 has identified resistance mutations that have been used to give a binary outcome of bNAb sensitivity, mirroring the role of genotyping for drug resistance [24,25,30^▪^]. However, as shown in a recent study by Gaebler *et al*. [18^▪▪^] compared to neutralisation assays, genotypic prediction only poorly identified resistant clones in prebNAb samples from participants who subsequently rebounded with 10-1074 resistant viruses. Of note, post-hoc neutralisation assays from participants with primary HIV infection recruited to a 10-1074 and 3BNC117 clinical trial [19^▪▪^], showed a correlation of baseline bNAb sensitivity with a better response to treatment. The improvements in next-generation sequencing and bioinformatics methods over the last few years have offered a range of *in silico* bNAb sensitivity prediction methods which may allow genotypic assays to become more predictive [38,39].

There are increasing numbers of computational models designed to predict inhibitory 10-1074 and 3BNC117 concentrations (IC50 and IC80) [29,40–46] based on genotypic data. The main limitation of these models lies in the short supply of neutralisation datasets to use for model training, especially for the very potent bNAbs like 3BNC117. This may explain the lower prediction performance of models when compared to the in-vivo response to bNAb treatment [41,42,45]. In addition, feature selection in most algorithms is primarily built upon genomic data of bNAb epitopes and only a few approaches incorporate additional features that influence bNAb binding, such as PNGs [45], or even regions outside the predefined epitope sites [42]. Interestingly, Meijers *et al.*[46] employed a bNAb resistance evolution model that factors in the fitness cost of resistance-associated mutations. The model was trained on a small dataset, but it suggested that viral escape from bNAbs can be predicted based on intra-host parameters (such as antibody levels and the degree of viraemia) rather than solely on resistance-conferring mutations.

Prediction accuracy notwithstanding, obtaining an adequate number of single HIV sequences that represent the full diversity of circulating viruses or latent proviral sequences is yet another challenge. Single genome HIV Env amplification and sequencing (using limiting dilution approaches) is the most commonly used method to do this, despite the long, laborious process and high cost [38]. Incorporating high-throughput elements in the assay, such as capture of single, intact viruses prior to amplification [47,48], would increase both the time and cost efficiency of the process. The combination of improved techniques to achieve high numbers of single genomes, greater sequencing read-length technologies, improved bioinformatic and machine learning approaches and larger, robust training datasets with clinical metadata are all key to developing a reproducible and accurate resistance-prediction methodology.

## CONCLUSION

There is growing evidence that bNAbs, such as 10-1074 and 3BNC117, are an exciting new treatment option which may be used alone or in conjunction with antiretroviral treatment to confer long-term virological control. Their use in hard-to-reach groups and regions with poor healthcare infrastructure could deliver viral suppression to individuals where it is currently challenging. However, viral resistance to bNAbs could set back the success of the intervention and so developing tools to accurately predict bNAb sensitivity has become a point of focus in the field. In this review, we outlined the signatures associated with resistance *in vivo* and the available tools for predicting sensitivity to 10-1074 and 3BNC117.However, as this is such a new area, knowledge of the virological genotypic and phenotypic features that predict resistance is still limited. As more clinical bNAb trials enrol, and more data are collected on outcomes, it will hopefully become possible to develop the necessary more robust methodologies needed to help guide clinical decision-making.

## Acknowledgements

*None*

### Financial support and sponsorship

*P.Z. and J.F. receive funding from the Bill and Melinda Gates Foundation (reference OPP1210792). M.A.A. is supported by a Sir Henry Dale Fellowship jointly funded by the Royal Society and Wellcome Trust (220171/Z/20/Z).*

### Conflicts of interest

*There are no conflicts of interest.*

## REFERENCES AND RECOMMENDED READING

Papers of particular interest, published within the annual period of review, have been highlighted as:

▪ of special interest
▪▪ of outstanding interest
